# Recognition Accuracy Using 3D Endoscopic Images for Superficial Gastrointestinal Cancer: A Crossover Study

**DOI:** 10.1155/2016/4561468

**Published:** 2016-08-15

**Authors:** Kosuke Nomura, Mitsuru Kaise, Daisuke Kikuchi, Toshiro Iizuka, Yumiko Fukuma, Yasutaka Kuribayashi, Masami Tanaka, Takahito Toba, Tsukasa Furuhata, Satoshi Yamashita, Akira Matsui, Toshifumi Mitani, Shu Hoteya

**Affiliations:** Department of Gastroenterology, Toranomon Hospital, Tokyo 105-8470, Japan

## Abstract

*Aim*. To determine whether 3D endoscopic images improved recognition accuracy for superficial gastrointestinal cancer compared with 2D images.* Methods*. We created an image catalog using 2D and 3D images of 20 specimens resected by endoscopic submucosal dissection. The twelve participants were allocated into two groups. Group 1 evaluated only 2D images at first, group 2 evaluated 3D images, and, after an interval of 2 weeks, group 1 next evaluated 3D and group 2 evaluated 2D images. The evaluation items were as follows: (1) diagnostic accuracy of the tumor extent and (2) confidence levels in assessing (a) tumor extent, (b) morphology, (c) microsurface structure, and (d) comprehensive recognition.* Results*. The use of 3D images resulted in an improvement in diagnostic accuracy in both group 1 (2D: 76.9%, 3D: 78.6%) and group 2 (2D: 79.9%, 3D: 83.6%), with no statistically significant difference. The confidence levels were higher for all items ((a) to (d)) when 3D images were used. With respect to experience, the degree of the improvement showed the following trend: novices > trainees > experts.* Conclusions*. By conversion into 3D images, there was a significant improvement in the diagnostic confidence level for superficial tumors, and the improvement was greater in individuals with lower endoscopic expertise.

## 1. Introduction

There have been major advances in the use of endoscopic devices for the diagnosis and treatment of gastrointestinal carcinoma as a result of new technology and the accumulation of cases that used these new technologies. However, information from conventional 2D images lacks information on depth perception, and, unlike real 3D information, 2D images provide merely probabilistic information. We believe that prediction with greater accuracy has become increasingly possible through experience, but we are not able to recognize tissue structures with 100% accuracy, and the potential for misrecognition of tissue structures still exists. Against this background, many institutions have begun to perform 3D endoscopic surgery in recent years. The utility of a 3D endoscopic system was reported in studies showing that the system improves the speed and accuracy of surgery through the enhancement of depth perception [1–3]. However, there have been few reports on endoscopic diagnosis to date [4]. In the field of diagnostic endoscopy, the incorporation of a 3D system is also expected to add an unprecedented amount of information on depth and improve the accuracy of diagnosis.

During the present study, we used ESD resection specimens to perform a comparison between 2D and 3D images to determine whether the accuracy of lesion recognition can be improved through the use of 3D endoscopic images.

## 2. Materials and Methods

### 2.1. Study Design

**Figure 1:**
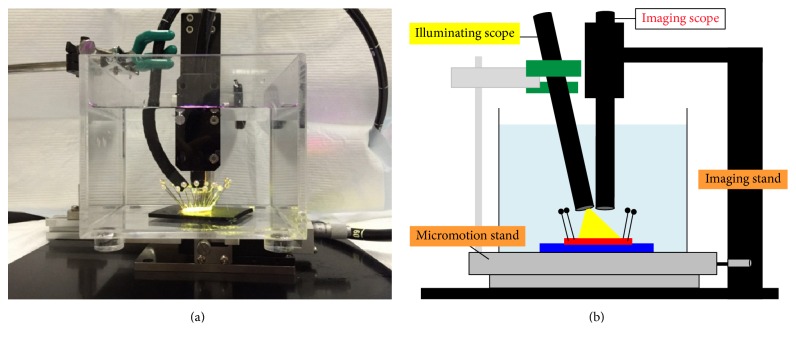
(a) The method of endoscopic imaging. (b) The simplified schematic of (a).

**Figure 2:**
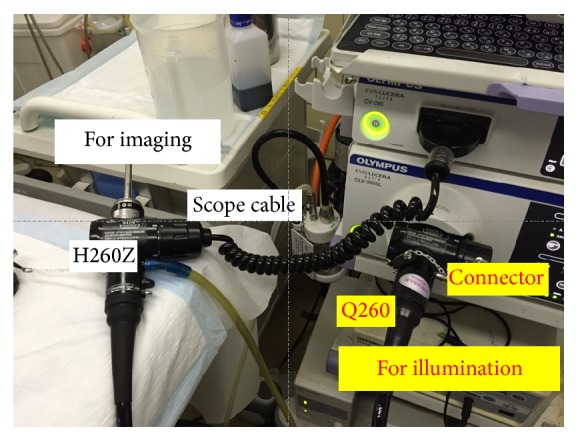
Connection method of the endoscopes.

**Figure 3:**
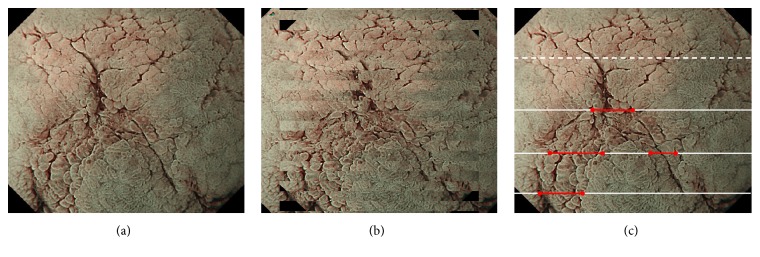
(a) A 2D sample of the resection specimen. (b) Example of parallax images (a 3D monitor and 3D glasses are required for three-dimensional visualization). (c) Adenocarcinoma components are detected in red line.

A total of 12 participants (4 novices, 4 trainees, and 4 experts) were recruited from a single institution, and 2 participants from each category were randomly allocated into two groups. An expert was defined as an endoscopist who had performed magnifying endoscopy for more than 500 cases, and a novice was defined as a physician who had never performed a single routine endoscopic examination. Test tissues were obtained from 20 ESD specimens: 15 superficial gastric cancers (14 cases of 0-IIc and 1 case of 0-IIb well-differentiated cancer), 3 superficial colon cancers (all IIa), and 2 superficial esophageal cancers (both 0-IIb). Each of the resected specimens was fixed on a black rubber plate with pins and immersed in a water box on a micromotion stage ((XCRS-80AR, MISUMI)). They were viewed under low magnification with Narrow Band Imaging system using a commercially available endoscopic unit (Evis Lucera Elite, Olympus Medical Systems, Co. Ltd., Tokyo Japan) and scopes fixed with a custom-made stand (Figures 1(a) and 1(b)). To create a 3D still image, parallax images were obtained by moving the fixed specimen in a horizontal direction with the micromotion stand. To acquire parallax images in the same lighting status, we used two scopes: one illumination scope simultaneously moved with the micromotion stage and another imaging scope did not move with it. A GIF-H260Z scope (Olympus Co., Japan) was used as the imaging scope and was attached to a scope cable, while a GIF-Q260 scope (Olympus Co., Japan) was used as the illuminating scope and was attached to a connector (Figure 2). Using an endoscopic image catalog containing two images, we extracted only odd rows from images for the left eye and even rows from images for the right eye and combined them to generate one line-by-line image. A 3D monitor and polarized 3D glasses enabled us to perceive a stereoscopic image through the observation of odd and even rows via the left and right eye, respectively. The two images on the retina were combined in the brain, which enabled perception of the virtual stereoscopic model (Figures 3(a) and 3(b)).

**Figure 4:**
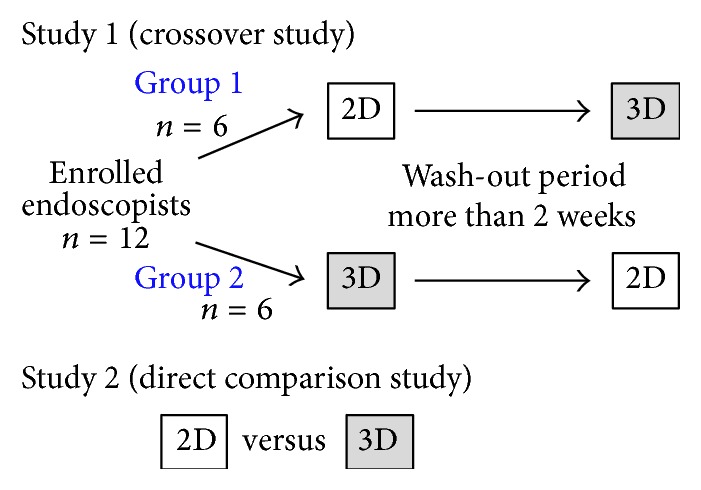
Flow diagram of the study.

Study 1: Figure 4 shows the study flow chart. Participants of group 1 evaluated only 2D images at first; then, after an interval of more than 2 weeks, they evaluated 3D images. Conversely, participants of group 2 first evaluated 3D images and after a set interval evaluated 2D images. The evaluation items were (1) diagnostic accuracy of tumor extent and (2) diagnostic confidence levels for the following items: (a) tumor extent, (b) morphology of the tumor surface, (c) microsurface structure (MSS) and/or microvascular structure (MVS), and (d) comprehensive recognition of the lesion. Study 2: the 2D and 3D images were examined sequentially, and improvement rates resulting from conversion into 3D images were evaluated in terms of confidence levels for items (a) to (d) above. In this study, we defined visual recognition of the demarcation line in the lesion as (a) tumor extent, recognition of irregular morphology in the lesion as (b) morphology of the tumor surface, recognition of MSS and/or MVS on the mucosal surface as (c) MSS and/or MVS, and recognition of (a)–(c) as a whole as (d) comprehensive recognition of the lesion.

When determining tumor extent, the test participant described a demarcation line on a printed image of each lesion. Calculations to determine accuracy were then made based on histological mapping (Figure 3(c)). Specifically, the total number of accurate diagnoses of lesions and nonlesions (as a numerator) was divided by the sum of the total number of starting and ending points (Figure 3(c), red lines) in the mapping image and the number of lines made in the nonlesion area (Figure 3(c), white solid lines) (as a denominator).

We evaluated confidence levels using a 5-point scale. In Study 1, the levels were as follows: 1 = extremely uncertain, 2 = uncertain, 3 = somewhat certain, 4 = almost certain, and 5 = absolutely certain. In Study 2, the levels were as follows: when compared to the 2D images, 1 = much more difficult to recognize clearly, 2 = somewhat more difficult to recognize clearly, 3 = the same as before (no change), 4 = somewhat easier to recognize clearly, and 5 = much easier to recognize clearly.

In the statistical analysis, numerical data and categorical data (5-point scale) were analyzed using the *t*-test and Wilcoxon signed-rank test, respectively. A *p* value of <0.05 was considered to be statistically significant.

## 3. Results

### 3.1. Study 1

#### 3.1.1. Effect of Examining 2D and 3D Images on Diagnostic Accuracy of Tumor Extent

Results are shown in Table 1. The diagnosis of tumor extent was accurate in 78.4% of all participants who viewed 2D images and in 81.1% of those who viewed 3D images, with a slight but not significant improvement in the diagnostic accuracy by examining 3D images. We found no significant differences when we compared group 1 (2D: 76.9%, 3D: 78.6%) and group 2 (2D: 79.9%, 3D: 83.6%), and the results were approximately equivalent. On examining the accuracy by participant skill level, the accuracy was slightly higher in the 3D group regardless of their skill levels, with no significant difference between the two groups.

#### 3.1.2. Effect of Examining 2D and 3D Images on Diagnostic Confidence Level

Results are shown in Table 2. Diagnostic confidence levels for all items ((a) to (d)) were higher for the 3D images compared with the 2D images with a significant difference observed in all cases: (a) 3.26 versus 3.70 (*p* < 0.01), (b) 3.24 versus 3.93 (*p* < 0.01), (c) 3.17 versus 3.58 (*p* < 0.01), and (d) 3.08 versus 3.60 (*p* < 0.01). Examining the confidence level for each item by observer skill level, we found that all experts had high confidence levels when viewing 3D images, with a significant difference observed for items (a) and (b): (a) 3.63 versus 3.85 (*p* < 0.05), (b) 3.93 versus 4.25 (*p* < 0.01), (c) 3.83 versus 3.90 (*p* = 0.292), and (d) 3.68 versus 3.87 (*p* = 0.054). Trainees showed higher confidence levels for all items ((a) to (d)) with a significant difference; the overall degrees of certainty were (a) 3.45 versus 3.70 (*p* < 0.05), (b) 3.33 versus 3.88 (*p* < 0.01), (c) 3.33 versus 3.60 (*p* < 0.05), and (d) 3.28 versus 3.66 (*p* < 0.05). Diagnostic confidence levels were also higher for novices for all items ((a) to (d)), with a significant difference in every item: (a) 2.71 versus 3.55 (*p* < 0.01), (b) 2.46 versus 3.68 (*p* < 0.01), (c) 2.35 versus 3.23 (*p* < 0.01), and (d) 2.29 versus 3.28 (*p* < 0.01). When we examined the average improvement in items (a) to (d) for the observers, experts showed a slight improvement (0.20 ± 0.81), but the trainees (0.37 ± 0.91) and novices (0.98 ± 0.98) showed greater improvement on average, indicating that improvement was greater for participants with a lower level of skill and significant differences were also observed (Table 3).

### 3.2. Study 2

Results are shown in Table 4. There was improvement in diagnostic confidence levels for items (a) to (d) for all specimens and the 95% confidence interval did not exceed a value of 3, so significant differences were observed. We also noted significant differences for all items ((a) to (d)) for experts, trainees, and novices when we examined the improvement by participant skill level.

## 4. Discussion

To date, 3D technology has been practically applied in various fields including movies, and applications in medical treatment have received significant attention in recent years, leading to an expansion in their use. In clinical settings, the application of 3D imaging has advanced from the perspectives of both three-dimensional image construction from investigation data and the implementation of stereoscopy. For the former, technology has allowed the creation of 3D images from data obtained from CT and MRI images, and this is reported to increase diagnostic ability and procedural precision [5–7]. In addition, the use of 3D printers has facilitated the practical application of materialized 3D data. Regarding implementation of stereoscopy in the latter, there have been several reports indicating utility in surgery, and use of 3D endoscopes facilitates stereoscopy [8–10], which suggests that techniques can be performed faster and more accurately. In the present report, we studied the utility of 3D images for the diagnosis of superficial GI tumor when using a flexible scope. This is the first report of its kind to date.

In this study, we found that when compared with 2D images, 3D images conferred a higher confidence level when determining tumor extent, for morphological recognition of MSS and MVS, when performing overall diagnosis, and particularly when identifying irregular morphology. These data indicate that converting images into 3D makes the surface morphology of superficial GI tumors more distinct, resulting in a concomitant increase in the accuracy of lesion recognition as 3D endoscope images provide depth perception.

In this study, the utility of conversion into 3D images was significantly greater for participants who had less experience with endoscopy. Experts could use their accumulated experience to visualize 3D structures from 2D images, whereas trainees and novices with little or no experience could not do so, resulting in a greater improvement after conversion into 3D imaging. These findings suggest that the training period for trainees and novices can be shortened by using 3D images. In addition, although significant differences were observed among experts during this crossover study, when compared to existing 2D images, slight improvement was observed in both the diagnostic accuracy and confidence level in lesion recognition. We believe that accuracy in recognition can be expected to improve by converting 2D images into 3D images, even for experts, because there was significant improvement during direct comparative studies.

Although no significant intergroup difference was observed in the diagnostic accuracy of tumor extent, we plan to reinvestigate the accuracy by increasing the number of specimens in future studies. The findings of this study also suggest that 3D visualization enables us to observe the features and morphology of lesions in greater detail, thereby contributing to the early detection of superficial cancer in clinical practice.

This study has some limitations. Subjective outcomes were used to investigate the utility of the 3D system. In principle, this study should have been blinded, but because of the obvious difference between 2D and 3D images, we conducted a crossover study instead. In addition, the sample size in this study was small as only 20 test lesions were examined, and thus the detection power for the differences between 2D and 3D images may be insufficient. In addition, 3D images and setting tested here are so artificial that the evidence obtained in this study is not directly applicable to clinical settings. We used ESD resection specimens and obtained only frontal still images. In the real world, lesions have blood supply and thus have a different color compared with resected specimens. Endoscopy views target lesions at various oblique angles. The present study used GI neoplastic lesions resected by ESD; thus we could not test the ability of 3D imaging in differentiating neoplasia from nonneoplasia. To overcome these limitations, we need to conduct clinical studies using 3D GI endoscopy. In the near future, we plan to perform studies using a prototype 3D endoscope for evaluating the impact of 3D imaging on diagnostic and therapeutic GI endoscopy.

## 5. Conclusions

The conversion from 2D into 3D imaging may improve the diagnostic confidence level for superficial GI neoplasia, and the improvement by 3D imaging is greater in individuals with lower endoscopic expertise. The development of flexible 3D endoscopy may be worthwhile to improve endoscopic diagnosis.

## Figures and Tables

**Table 1:** Average accuracy in determining the extent of disease.

	2D (%)	3D (%)	*p*
All observers	78.4 ± 27.7	81.1 ± 25.5	0.177
Group 1	76.9 ± 27.6	78.6 ± 27.5	0.499
Group 2	79.9 ± 27.6	83.6 ± 23.1	0.079
Experts	80.8 ± 25.9	84.4 ± 23.5	0.061
Trainees	80.3 ± 26.3	81.4 ± 25.0	0.604
Novices	74.2 ± 30.0	77.4 ± 27.4	0.319

**Table 2:** The degree of certainty of Study 1 (average ± SD).

	(a) Tumor extent			(b) Morphology of tumor surface			(c) MSS or/and MVS			(d) Comprehensive recognition		
	2D	3D	*p*	2D	3D	*p*	2D	3D	*p*	2D	3D	*p*
All observers	3.26 ± 1.20	3.70 ± 1.00	<0.01	3.24 ± 1.18	3.93 ± 1.03	<0.01	3.17 ± 1.14	3.58 ± 1.01	<0.01	3.08 ± 1.15	3.60 ± 0.96	<0.01
Experts	3.63 ± 1.18	3.85 ± 1.07	<0.05	3.93 ± 0.95	4.25 ± 0.87	<0.01	3.83 ± 1.00	3.90 ± 0.98	0.180	3.68 ± 1.06	3.87 ± 0.96	0.064
Trainees	3.45 ± 1.12	3.70 ± 0.98	<0.05	3.33 ± 1.03	3.88 ± 1.12	<0.01	3.33 ± 0.91	3.60 ± 0.82	<0.05	3.28 ± 1.04	3.66 ± 0.85	<0.01
Novices	2.71 ± 1.12	3.55 ± 0.92	<0.01	2.46 ± 1.05	3.68 ± 1.00	<0.01	2.35 ± 0.96	3.23 ± 1.10	<0.01	2.29 ± 0.87	3.28 ± 0.97	<0.01

**Table 3:** Average improvement of Study 1 (average ± SD).

	(a) Tumor extent	(b) Morphology of tumor surface	(c) MSS or/and MVS	(d) Comprehensive recognition	Average
Experts	0.23 ± 0.92	0.33 ± 0.80	0.08 ± 0.63	0.19 ± 0.85	0.20 ± 0.81^*∗*^
Trainees	0.25 ± 0.94	0.55 ± 0.97	0.28 ± 0.82	0.39 ± 0.84	0.37 ± 0.91^*∗*,*∗∗*^
Novices	0.84 ± 1.03	1.21 ± 1.21	0.88 ± 0.99	0.99 ± 0.96	0.98 ± 0.98^*∗∗*^

^*∗*^ *p* < 0.05. ^*∗∗*^ *p* < 0.01.

**Table 4:** The degree of certainty of Study 2.

	(a) Tumor extent		(b) Morphology of tumor surface		(c) MSS or/and MVS		(d) Comprehensive recognition	
	Average	95% CI	Average	95% CI	Average	95% CI	Average	95% CI
All observers	3.48	3.40–3.56	4.07	3.99–4.15	3.32	3.24–3.40	3.53	3.41–3.63
Experts	3.31	3.18–3.44	3.95	3.84–4.06	3.15	3.05–3.25	3.48	3.35–3.61
Trainees	3.51	3.38–3.64	4.03	3.88–4.18	3.20	3.11–3.29	3.48	3.33–3.57
Novices	3.61	3.46–3.76	4.23	4.09–4.36	3.61	3.44–3.78	3.64	3.48–3.80
